# Transcriptomics Reveals Discordant Lipid Metabolism Effects between In Vitro Models Exposed to Elafibranor and Liver Samples of NAFLD Patients after Bariatric Surgery

**DOI:** 10.3390/cells11050893

**Published:** 2022-03-04

**Authors:** Joost Boeckmans, Alexandra Gatzios, Anja Heymans, Matthias Rombaut, Vera Rogiers, Joery De Kock, Tamara Vanhaecke, Robim M. Rodrigues

**Affiliations:** Department of In Vitro Toxicology and Dermato-Cosmetology, Faculty of Medicine and Pharmacy, Vrije Universiteit Brussel, 1090 Brussels, Belgium; alexandra.gatzios@vub.be (A.G.); anja.heymans@vub.be (A.H.); matthias.rombaut@vub.be (M.R.); vera.rogiers@vub.be (V.R.); joery.de.kock@vub.be (J.D.K.); tamara.vanhaecke@vub.be (T.V.)

**Keywords:** NASH, NAFLD, elafibranor, in vitro, hSKP-HPC, HepaRG, hepatocytes, bariatric surgery, transcriptomics

## Abstract

Background and aims: Non-alcoholic steatohepatitis (NASH) is a life-threatening stage of non-alcoholic fatty liver disease (NAFLD) for which no drugs have been approved. We have previously shown that human-derived hepatic in vitro models can be used to mimic key cellular mechanisms involved in the progression of NASH. In the present study, we first characterize the transcriptome of multiple in vitro NASH models. Subsequently, we investigate how elafibranor, which is a peroxisome proliferator-activated receptor (PPAR)-α/δ agonist that has recently failed a phase 3 clinical trial as a potential anti-NASH compound, modulates the transcriptome of these models. Finally, we compare the elafibranor-induced gene expression modulation to transcriptome data of patients with improved/resolved NAFLD/NASH upon bariatric surgery, which is the only proven clinical NASH therapy. Methods: Human whole genome microarrays were used for the transcriptomics evaluation of hepatic in vitro models. Comparison to publicly available clinical datasets was conducted using multiple bioinformatic application tools. Results: Primary human hepatocytes (PHH), HepaRG, and human skin stem cell-derived hepatic progenitors (hSKP-HPC) exposed to NASH-inducing triggers exhibit up to 35% overlap with datasets of liver samples from NASH patients. Exposure of the in vitro NASH models to elafibranor partially reversed the transcriptional modulations, predicting an inhibition of toll-like receptor (TLR)-2/4/9-mediated inflammatory responses, NFκB-signaling, hepatic fibrosis, and leukocyte migration. These transcriptomic changes were also observed in the datasets of liver samples of patients with resolved NASH. Peroxisome Proliferator Activated Receptor Alpha (PPARA), PPARG Coactivator 1 Alpha (PPARGC1A), and Sirtuin 1 (SIRT1) were identified as the major common upstream regulators upon exposure to elafibranor. Analysis of the downstream mechanistic networks further revealed that angiopoietin Like 4 (ANGPTL4), pyruvate dehydrogenase kinase 4 (PDK4), and perilipin 2 (PLIN2), which are involved in the promotion of hepatic lipid accumulation, were also commonly upregulated by elafibranor in all in vitro NASH models. Contrarily, these genes were not upregulated in liver samples of patients with resolved NASH. Conclusion: Transcriptomics comparison between in vitro NASH models exposed to elafibranor and clinical datasets of NAFLD patients after bariatric surgery reveals commonly modulated anti-inflammatory responses, but discordant modulations of key factors in lipid metabolism. This discordant adverse effect of elafibranor deserves further investigation when assessing PPAR-α/δ agonism as a potential anti-NASH therapy.

## 1. Introduction

Non-alcoholic fatty liver disease (NAFLD) is an umbrella term that covers a range of liver pathologies, starting from uncomplicated hepatic steatosis to more severe disease stadia, including non-alcoholic steatohepatitis (NASH), fibrosis, cirrhosis, and ultimately hepatocellular carcinoma [1]. NASH is characterized by hepatic fat accumulation and inflammation and affects approximately 5 percent of the global population [1,2]. It is considered as the tipping point to the most severe stages of NAFLD and also carries high risk of (extra)hepatic complications [1,2]. Despite the urgent medical need, no drugs have been approved yet to treat NASH [3]. Weight loss through lifestyle intervention and bariatric surgery are still the only options to treat NASH [4,5]. Multiple phase 2 and 3 clinical studies are ongoing, but recently several phase 3 trials failed to meet their primary endpoints. This indicates that more performant and predictive preclinical models should be employed during the investigation of novel anti-NASH therapeutics. We recently showed that human hepatic in vitro models, including primary human hepatocytes (PHH), human skin precursor-derived hepatic progenitor cells (hSKP-HPC), and HepaRG and HepG2 cell lines, can model key NASH-specific cellular mechanisms and capture potential anti-NASH properties of novel compounds such as peroxisome proliferator-activated receptor (PPAR) agonists, a drug class that is under clinical evaluation for anti-NASH treatment [6,7]. PPARs have a regulatory role in hepatic lipid metabolism and inflammation and multiple dual- and pan-PPAR agonists, which can supposedly lead to a reduction of hepatic lipids and inflammation, are under investigation, including lanifibranor (a pan-PPAR agonist, phase 3, NCT04849728), saroglitazar (a dual PPAR-α/γ agonist, phase 2, NCT03863574), and elafibranor (a dual PPAR-α/δ agonist, phase 3, NCT02704403) [3]. Despite promising data obtained in early clinical trials, it was recently reported in an interim analysis of the phase 3 ‘RESOLVE-IT’ trial that elafibranor did not meet its primary endpoint, namely ‘NASH resolution without worsening fibrosis’ [8,9]. However, no mechanistic explanation for the failed trial was provided. Since no clinical data (including transcriptomics data) or study samples are available from NASH patients treated with elafibranor, we were prompted to perform whole genome transcriptomics analyses of human-based in vitro NASH models exposed to elafibranor in order to investigate its molecular effects. By comparing the obtained results to transcriptomics datasets of liver samples from NAFLD patients after bariatric surgery, we aimed at gaining insights in the mechanisms that could possibly lay at the basis of the negative outcome of the interim analysis of the phase 3 ‘RESOLVE-IT’ trial. Bariatric surgery impacts several hepatic metabolic pathways even before weight loss occurs, leading to a reduction of liver triglyceride levels as a major cause for the reduction of NASH [10,11]. Therefore, it was considered as a valid reference.

## 2. Materials and Methods

### 2.1. hSKP-HPC Cell Cultures

hSKP were isolated from foreskin samples after circumcision of 2–10 year old boys after obtaining informed consent from the parents and authorization from the medical ethical committee of the UZ Brussel. hSKP were isolated, cryopreserved, cultured, and differentiated to hSKP-HPC as earlier described [12]. Briefly, hSKP were recovered from liquid nitrogen at passage 6 and plated onto 75 cm^2^ culture flasks (BD Falcon, Erembodegem, Belgium) in a humidified incubator with 5% (*v/v*) CO_2_ at 37 °C. Hereafter, hSKP were split twice using TrypLE Express reagent (Thermo Fisher Scientific, Waltham, MA, USA). Hepatic differentiation was initiated when the culture reached 90 to 95% confluence in rat tail collagen type I (Corning Incorporated, Corning, NY, USA)-coated 75 cm² culture flasks (BD Falcon, Erembodegem, Belgium) in a humidified incubator with 5% (*v/v*) CO_2_ at 33 °C. At day 20, the cells were detached using TrypLE Express reagent (Thermo Fisher Scientific, Waltham, MA, USA), transferred onto rat tail collagen type I (Corning Incorporated, Corning, NY, USA)-coated 24-multiwell plates (BD Falcon, Erembodegem, Belgium), and kept in culture for 7 more days. Then, hSKP-HPC were exposed for 24 h in a humidified incubator with 5% (*v/v*) CO_2_ at 37 °C to ‘NASH’-inducing factors, consisting of 100 nM insulin, 65 µM sodium oleate, 45 µM palmitic acid, 4.5 mg/mL glucose (all Sigma-Aldrich, St. Louis, MO, USA), 50 ng/mL tumor necrosis factor (TNF)-α (Prospec, Rehovot, Israel), 25 ng/mL interleukin (IL)-1β, and 8 ng/mL transforming growth factor (TGF)-β (both Peprotech, Cranbury, NJ, USA), as earlier documented [6].

### 2.2. HepaRG Cell Cultures

Differentiated HepaRG cells were purchased from Biopredic International (St.-Grégoire, France) and cultured according to the supplier’s instructions. Briefly, the cryopreserved differentiated HepaRG cells were plated onto rat tail collagen type I (Corning Incorporated, Corning, NY, USA)-coated 24-multiwell plates (BD Falcon, Erembodegem, Belgium) using basal hepatic medium (Biopredic International, St.-Grégoire, France) supplemented with ‘thawing/plating/general purpose medium supplement with antibiotics’ (Biopredic International, St.-Grégoire, France) in a humidified incubator with 5% (*v/v*) CO_2_ at 37 °C. After 24 h, the medium was replaced by basal hepatic medium (Biopredic International, St.-Grégoire, France) supplemented with ‘maintenance/metabolism medium supplement with antibiotics’ (Biopredic International, St.-Grégoire, France) and changed every 2–3 days for one week. ‘NASH’ inducers were added to William’s E medium (Thermo Fisher Scientific, Waltham, MA, USA) supplemented with 10% (*v/v*) FBS (Hyclone Laboratories, Logan, UT, USA), 1% (*v/v*) PenStrep (Thermo Fisher Scientific, Waltham, MA, USA), 2 mM L-glutamine (Sigma-Aldrich, St. Louis, MO, USA), 872.69 nM insulin (Sigma-Aldrich, St. Louis, MO, USA), and 0.5 nM hydrocortisone. The ‘NASH’ inducers were identical to those described under ‘hSKP-HPC culture’, with the exception of the insulin concentration, which was raised to 8726.92 nM.

### 2.3. Primary Human Hepatocyte Cell Cultures

Fresh primary human hepatocytes (PHH) cultures were purchased from Biopredic International (St.-Grégoire, France). The cells were isolated and plated freshly onto rat tail collagen type I-coated 24-multiwell plates. After isolation, the cells were maintained in long term culture medium (Biopredic International, St.-Grégoire, France) and shipped at 37 °C. Four days after isolation, the cells were exposed for 24 h to conditions identical to those described for HepaRG cell cultures.

### 2.4. Compound Preparation

Elafibranor (Adooq Bioscience, Irvine, CA, USA) was dissolved in dimethylsulfoxide (DMSO) (Sigma–Aldrich, St. Louis, MO, USA) at a concentration of 60 mM. Elafibranor was added to the cultures at 60 µM concomitantly with the ‘NASH’ triggers.

### 2.5. RNA Extraction

After exposure for 24 h, the cell cultures were lysed using RNA-lysis buffer containing 1% *v/v* β-mercaptoethanol (Sigma-Aldrich, St. Louis, MO, USA) and stored in RNAse-free tubes at −80 °C. Total RNA was extracted and purified using the GenElute Mammalian Total RNA Purification Miniprep Kit (Sigma-Aldrich, St. Louis, MO, USA). RNA quantification was performed using a Nanodrop spectrophotometer (Thermo Fisher Scientific, Waltham, MA, USA).

### 2.6. Microarray

Whole genome microarrays were run using GeneChip™ Human Genome U133 Plus 2.0 Arrays (Thermo Fisher Scientific, Waltham, MA, USA) and reagents. 50 to 100 ng RNA was amplified using the GeneChip 3′ IVT PLUS Reagent Kit. aRNA was purified using magnetic particles before fragmentation. Fragmented aRNA was hybridized to Affymetrix Human Genome U133 plus 2.0 arrays and put in a Genechip Hybridization Oven-645 (Thermo Fisher Scientific, Waltham, MA, USA) at 45 °C and rotated at 60 rpm for 16 h. Then, the arrays were washed using a Genechip Fluidics Station-450 (Thermo Fisher Scientific, Waltham, MA, USA) and stained with an Affymetrix HWS kit. The chips were scanned using a GeneChip™ Scanner 3000 7G (Thermo Fisher Scientific, Waltham, MA, USA). Background adjustment, normalization (quantile), and summarization (median polish) of the raw data was done using Robust Multichip Average (RMA) Express. Volcano plots of all probesets (a probeset represents one or multiple hybridization probe pairs used together to interrogate a sequence corresponding to a specific gene) were made using Transcriptome Analysis Console (TAC) software (Thermo Fisher Scientific, Waltham, MA, USA) (eBayes, FDR fold change 2 and *p* < 0.025). Pathway analyses were performed using Ingenuity Pathway Analysis (IPA) (Qiagen, Hilden, Germany, version summer release 2020). A two-sided t-test with unequal variance was used for calculating the *p*-values before loading the data in IPA. Genes with a fold change <−2 or >+2 and *p* < 0.025 were included for further analysis. Activation z-scores of ≥+2 and ≤−2 were considered as significant for a likely activation or inhibition status, respectively. All specific terms related to IPA and used in the manuscript have been explained in Table S1. The raw .CEL files and normalized data of PHH (control-‘NASH’-‘NASH’ + elafibranor), HepaRG (‘NASH’-‘NASH’ + elafibranor), and hSKP-HPC (‘NASH’ + elafibranor) have been deposited in the Gene Expression Omnibus (GEO) database with accession number GSE166186.

### 2.7. Publicly Available Datasets

Microarray datasets of HepaRG control samples were obtained from Rodrigues et al. (GSE74000) [13]. Microarray datasets of hSKP-HPC control samples and hSKP-HPC ‘NASH’ samples were obtained from Boeckmans et al. (GSE126484) [6]. For comparative pathway analysis, IPA-deposited transcriptomics analyses (accessed October 2020–April 2021) of human liver biopsies were assessed in IPA-Analysis Match (Analysis names, disease states, dataset IDs, contributors, and references: ‘3-obesity [liver] NA 9506′ (obesity (n = 16) vs. normal control (n = 12), GSE48452, Ahrens et al. [14]), ‘1- fatty liver [liver] NA 9504′ (steatosis (n = 9) vs. normal control (n = 12), GSE48452, Ahrens et al. [14]), ‘2-nonalcoholic steatohepatitis (NASH) [liver] NA 9505’ (NASH (n = 17) vs. normal control (n = 12), GSE48452, Ahrens et al. [14]), ‘3-nonalcoholic steatohepatitis (NASH) [liver] NA 3543’(NASH (n = 7) vs. normal control (n = 19), E-MEXP-3291, Lake et al. [15]), ‘2-nonalcoholic steatohepatitis (NASH) [liver] NA 10520’ (NASH (n = 24) vs. normal control (n = 37), GSE61260, Horvath et al. [16]), ‘2-nonalcoholic steatohepatitis (NASH) [liver] NA 2178’ (NASH (n = 15) vs. normal control (n = 14), GSE126848, Suppli et al. [17]), ‘5-obesity [liver] NA 9508’ (obesity + bariatric surgery (n = 11) vs. none (n = 4), GSE48452, Ahrens et al. [14]), ‘4-fatty liver [liver] NA 9507’ (steatosis + bariatric surgery (n = 5) vs. none (n = 7), GSE48452, Ahrens et al. [14]), ‘1-nonalcoholic fatty liver disease (NAFLD); overweight [liver] NA 12329’ (NAFLD + bariatric surgery (n = 23) vs. none (n = 28), GSE83452, Lefebvre et al. and ‘3-nonalcoholic steatohepatitis (NASH);overweight [liver] NA 12331’ (NASH + bariatric surgery (n = 15) vs. baseline (n = 55), GSE83452, Lefebvre et al. [18])).

### 2.8. Statistical Analysis

Z-scores were calculated using IPA. Other statistical analyses were performed using GraphPad Prism 7.0 (San Diego, CA, USA). The data are presented as the mean ± standard deviation. A one-way ANOVA with post hoc Bonferroni correction was used when comparing selected groups (control with ‘NASH’ and ‘NASH’ with ‘NASH’ + elafibranor). Three independent replicates were used.

## 3. Results

### 3.1. Transcriptomes of Human Hepatic In Vitro NASH Models Exhibit Similarities with Those of Human NASH Liver

**Figure 1:**
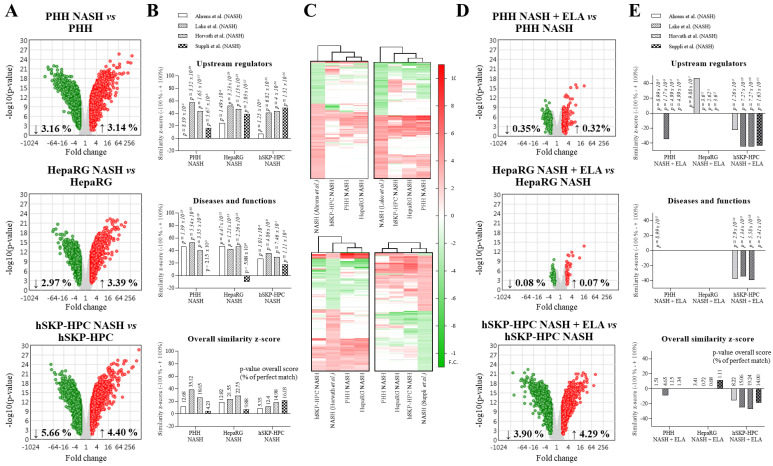
Transcriptional responses of human hepatic in vitro models exposed to ‘NASH’ triggers correspond with those of human NASH liver biopsies. (**A**) Microarray volcano plots of differentially modulated probesets in PHH, HepaRG, and hSKP-HPC cultures exposed to ‘NASH’ triggers. (**B**) Similarity analysis (IPA z-score) of in vitro NASH models and human liver NASH datasets. (**C**) Hierarchical clustering of upstream regulators in the in vitro NASH models with human liver NASH datasets. (**D**) Volcano plots of probesets of PHH-, HepaRG- and hSKP-HPC-‘NASH’ cultures exposed to elafibranor. (**E**) Similarity analysis (IPA z-score) of in vitro NASH models exposed to elafibranor with human liver NASH datasets [Volcano plots exclusion criteria: eBayes, FDR fold change <−2 or >+2 and *p* < 0.025; IPA analysis: Fisher’s Exact Test, fold change cutoff: <−2 or >+2 and *p* < 0.025].

Exposure of PHH, HepaRG, and hSKP-HPC cultures to ‘NASH’ triggers results in total probeset modulations of 6.30 %, 6.36 %, and 10.06 % in TAC, respectively, indicating that hSKP-HPC most sensitively responds to steatogenic and inflammatory triggers (Figure 1A, Figure S1). These transcriptional alterations result in 2001, 2277, and 3213 analysis-ready molecules/genes for PHH, HepaRG, and hSKP-HPC in IPA, respectively. Comparative analysis of ‘upstream regulators’ (URs) and ‘diseases and functions’ between the in vitro datasets and NASH patient datasets reveals up to 50% similarity, depending on the model used and the reference clinical dataset, and shows NASH-specific transcriptional alterations in the in vitro models exposed to ‘NASH’ triggers (Figure 1B, top panels). NASH-triggered PHH exhibits the highest overall similarity z-score (with the dataset of Lake et al.), suggesting that PHH most closely mimics this patient-specific transcriptome (Figure 1B). Hierarchical clustering of URs of the 4 NASH patient datasets with the in vitro ‘NASH’ datasets shows that HepaRG cultures are closer to PHH cultures than hSKP-HPC cultures (Figure 1C top and bottom panels). NASH-triggered hSKP-HPC are, however, closer to the patient dataset of Suppli et al. than to the PHH and HepaRG in vitro NASH models (Figure 1C bottom panel). This indicates that all three in vitro NASH models exhibit a number of NASH-specific transcriptional features. Exposure of the in vitro NASH models to elafibranor results in modulation of 0.67%, 0.15%, and 8.19% of probesets in PHH, HepaRG, and hSKP-HPC, respectively, indicating that the latter model most sensitively responds to this compound (Figure 1D and Figure S2). Similarity analysis of elafibranor-exposed PHH, HepaRG, and hSKP-HPC (based on 106, 45, and 2857 analysis-ready molecules/genes, respectively) indicates reversion of NASH-specific transcriptional responses, which is the most pronounced for hSKP-HPC (Figure 1E).

### 3.2. Elafibranor Reduces Toll-like Receptor-Dependent Inflammation in PHH and hSKP-HPC In Vitro NASH Models

**Figure 2:**
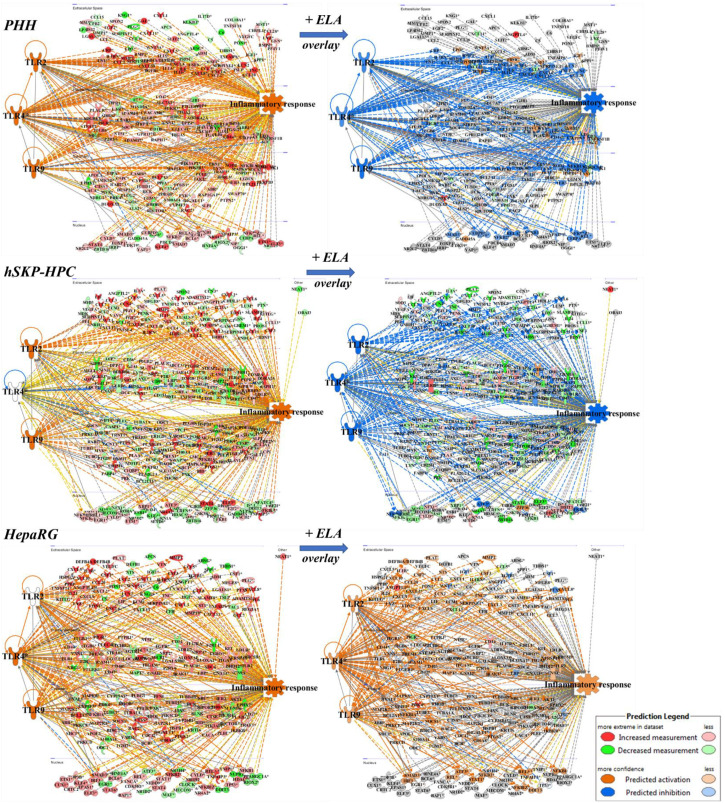
Pathway analysis predicts a reduction of TLR-2/4/9-dependent inflammatory responses by elafibranor in PHH and hSKP-HPC ‘NASH’ cultures, but not in HepaRG ‘NASH’ cultures. Prediction chart based on TLR signaling shows an activation of inflammatory responses in PHH, hSKP-HPC and HepaRG cell cultures exposed to NASH triggers (left panels). When PHH and hSKP-HPC ‘NASH’ cultures are further exposed to elafibranor, this inflammatory response is inhibited in PHH and hSKP-HPC cell cultures, but not in HepaRG cultures (right panels) [orange line = ‘leads to activation’; blue line = ‘leads to inhibition’; yellow line = ‘findings inconsistent with state of downstream molecule’; grey line = ‘effect not predicted’].

One specific mechanism by which hepatic inflammation is initiated in NASH occurs via Toll-like receptors (TLRs) and MyD88 signaling [19]. Free fatty acids, such as palmitic acid, and damage-associated molecular patterns (DAMPs), such as high mobility group box 1 (HMGB1), are able to activate TLRs to initiate the inflammatory response. Therefore, we investigated the involvement of TLR-2, -4, and -9 signaling on the inflammatory response in the in vitro models exposed to NASH triggers and elafibranor. Pathway analysis shows that TLR-2, -4, and -9 signaling are activated in NASH-triggered PHH, but only TLR-2 and -9 or TLR-4 and -9 contribute to the inflammatory response in HepaRG and hSKP-HPC, respectively (Figure 2). These data suggest that the NASH-triggered hepatic in vitro models exhibit subtle differences in inflammation-promoting mechanisms. However, exposure to elafibranor seems to attenuate the inflammatory response by intervening in TLR-2, -4 as -9 signaling in PHH and hSKP-HPC (Figure 2). This effect is, however, absent in elafibranor-exposed HepaRG ‘NASH’ cultures, which do not show inhibition of TLR signaling, nor inhibition of the inflammatory response.

### 3.3. Similarities in NASH-Related Upstream Regulators and Canonical Pathways in Hepatic In Vitro NASH Models Exposed to Elafibranor and Liver Samples of Patients after Bariatric Surgery

**Figure 3:**
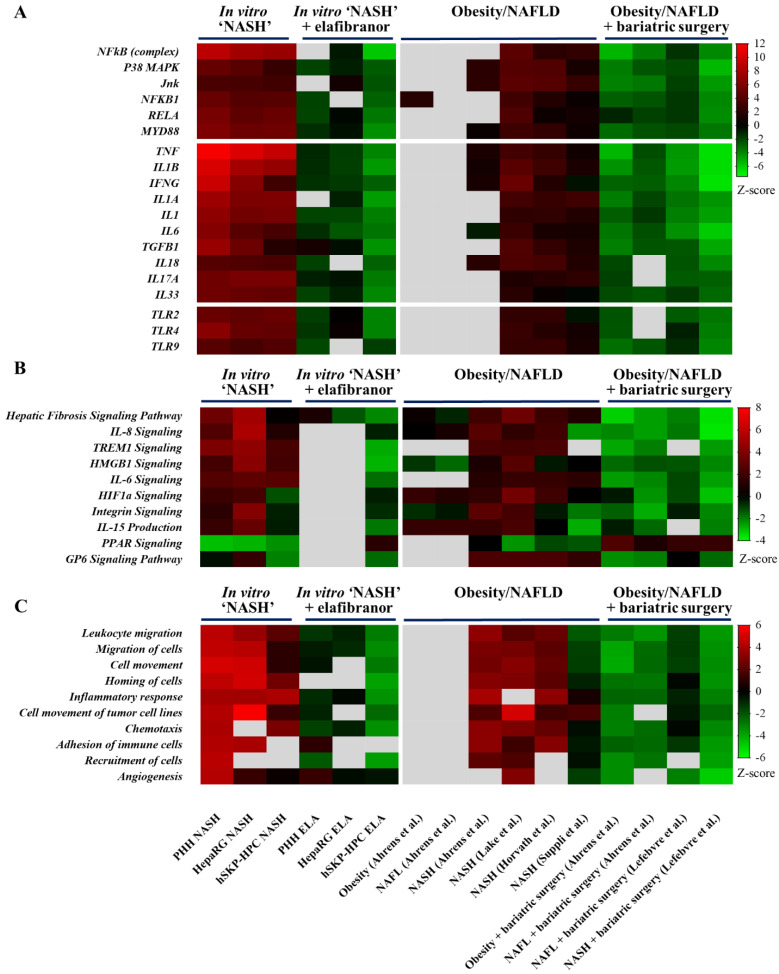
Prediction z-scores of NASH-specific upstream inflammatory regulators, canonical pathways, and inflammatory functions in hepatic in vitro NASH models exposed to elafibranor show a similar modulation to liver samples of patients with resolved NAFLD by bariatric surgery. Microarray analysis of upstream inflammatory mediators and Toll-like receptors (**A**), canonical pathways (**B**), and inflammatory functions (**C**) in PHH, HepaRG, and hSKP-HPC cultures exposed to ‘NASH’ triggers in the presence or absence of elafibranor is compared to data sets from NASH patients and NASH patients that underwent bariatric surgery [z-score of ≥2 indicates a significant activation; z-score of ≤−2 indicates a significant inhibition; grey fields indicate no available z-score].

Z-score determination of URs in NASH-triggered PHH, HepaRG, and hSKP-HPC cultures shows the predicted activation of prototypical mediators of inflammation during NASH, which is the most pronounced for NFkB (complex). However, p38 (mitogen-activated protein kinase) MAPK and Jun are also predicted as being activated in all three in vitro NASH models, indicating that multiple mechanisms lay at the basis of inflammatory signaling. Of note, these mediators are also predicted as being activated in the NASH patient datasets, indicating the clinical relevance of the inflammatory response in the in vitro NASH models at the transcriptional level. Inflammatory cytokines that lay at the origin of inflammation in human NASH are also highly involved in the in vitro NASH datasets, suggesting that the activation of the inflammatory transcription factors and kinases also occurs in a similar way. The TLR and MyD88 signaling are also activated in both in vitro and patient datasets. Of note, these NASH-inducing transcriptional alterations that occur in vitro can only be observed in specific NASH patient datasets, and not in datasets derived from livers obtained from patients suffering from obesity or steatosis alone. Exposure of the NASH-triggered hepatic in vitro models to elafibranor reverses the prediction statuses of most URs, which is the least pronounced for the HepaRG cultures. Furthermore, the elafibranor-induced reversion of prediction statuses of NASH-specific URs also occurs in the livers of patients that underwent bariatric surgery, which suggests that some of the weight loss-induced transcriptional effects can be observed in vitro through pharmacological intervention (Figure 3A).

Exposure of PHH, HepaRG, and hSKP-HPC to ‘NASH’ triggers results in predicted activation of NASH-specific canonical pathways, including HMGB1 and triggering receptor expressed on myeloid cells-1 (TREM1)-signaling that are predicted as being activated in all three in vitro NASH datasets and at least one NASH patient dataset (Figure 3B). These canonical pathways are only predicted as being inhibited in the elafibranor-treated hSKP-HPC NASH samples, yet also in liver samples obtained from patients that underwent bariatric surgery, pointing to the clinical relevance of the latter model. Further, PPAR-signaling is impaired in all in vitro NASH datasets, which is also seen in human NASH liver samples. However, a recovery of the PPAR signaling is only observed in hSKP-HPC NASH model exposed to elafibranor and the clinical datasets from patients with resolved NASH upon bariatric surgery (Figure 3B).

Furthermore, NASH-triggered PHH, HepaRG, and hSKP-HPC were found to exhibit increased prediction statuses of functions related to chemotaxis and inflammatory cell migration (Figure 3C). These prominent inflammatory responses can be partially ascribed to the increased mRNA levels of a series of inflammatory chemokines (Figure S3). The cell migration-promoting functions that are activated in the in vitro models are also found as being activated in the NASH patient liver samples, suggesting that the similarities between the in vitro and patient datasets mainly rely on cell migratory processes. The addition of elafibranor to NASH-stimulated cell cultures robustly induces negative prediction statuses of these functions only in hSKP-HPC, which indicates that this model most sensitively responds to this compound to alter NASH-specific disease mechanisms. Moreover, these alterations are also present in liver biopsies obtained from patients with resolved NAFLD upon undergoing bariatric surgery, which further illustrates the relevance of hSKP-HPC.

### 3.4. PPARGC1A, PPARA, and SIRT1 Regulate Shared Elafibranor-Induced Genes in NASH-Triggered PHH, HepaRG, and hSKP-HPC

**Figure 4:**
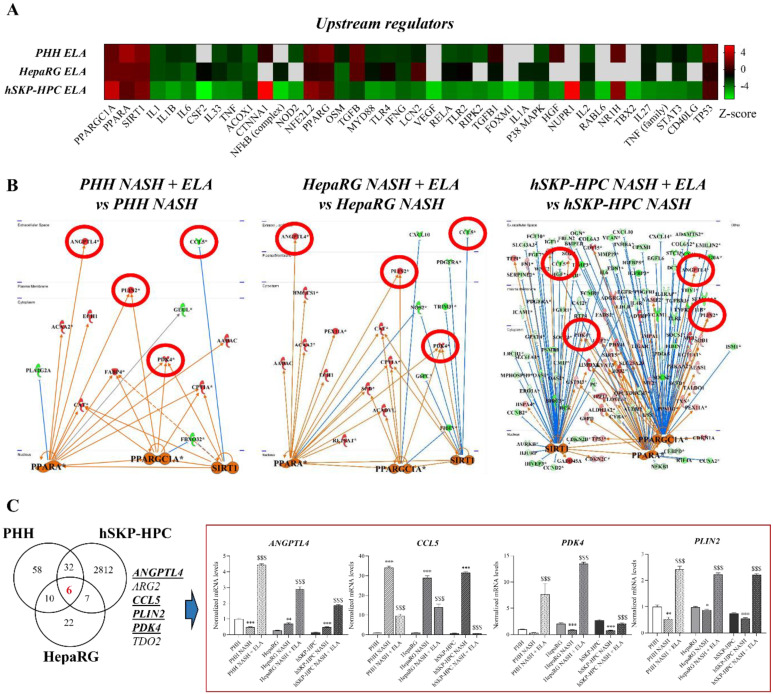
PPAR-α/δ-induced commonly modulated genes among hepatic in vitro NASH models are regulated by shared upstream regulators. (**A**) Top-upstream regulators in PHH, HepaRG, and hSKP-HPC NASH models exposed to elafibranor. (**B**) Mechanistic networks of hepatic in vitro NASH models exposed to elafibranor with connected SIRT1, PPARGC1A1, and PPARA top upstream regulators. (**C**) Number of commonly modulated genes induced by elafibranor in PHH-, HepaRG-, and hSKP-HPC NASH models. (**A**,**B**): fold change: <−2 or >+2 and Fisher’s Exact Test with *p* < 0.025; (**C**) One-way ANOVA with post hoc Bonferroni correction vs. control condition (*, ** and ***; *p* ≤ 0.05, *p* ≤ 0.01, and *p* ≤ 0.001) and vs. ‘NASH’ condition ($$$; *p* ≤ 0.001)].

In order to detect biologically relevant changes in gene expression in response to elafibranor, we opted to combine the data of the three different human-based cell systems (PHH, HepaRG, and hSKP-HPC). Comparison analysis of NASH-triggered PHH, HepaRG, and hSKP-HPC cultures exposed to elafibranor unveils that Peroxisome Proliferator Activated Receptor Alpha (PPARA), PPARG Coactivator 1 Alpha (PPARGC1A), and Sirtuin 1 (SIRT1) are the highest expressed common upstream regulators, suggesting that this triad of regulators control the expression of an important subset of genes involved in PPAR-α/δ signaling (Figure 4A). The mechanistic networks of PPARGC1A, PPARA, and SIRT1 and their downstream modulators based on the datasets of PHH-, HepaRG-, and hSKP-HPC- NASH exposed to elafibranor, unveils common regulation of CCL5, ANGPTL4, PDK4, and PLIN2 (Figure 4B). In addition, an unfiltered comparison analysis of the datasets obtained from the three in vitro NASH models exposed to elafibranor shows that elafibranor commonly modulates 6 genes, namely TDO2, ARG2, and also the previously identified CCL5, ANGPTL4, PDK4, and PLIN2 (Figure 4C). Considering that the level of expression of TDO2 is very low in hSKP-HPC and that the modulations of ARG2 are not consistent among the different in vitro models (Figure S4), only CCL5, ANGPTL4, PDK4, and PLIN2 are regulated by shared top-upstream regulators, and we selected these 4 genes for further investigation.

### 3.5. Elafibranor-Mediated Induction of ANGPTL4, PDK4, and PLIN2 In Vitro Predicts Induction of Steatosis, Contrarily to Anti-NASH Bariatric Surgery

**Figure 5:**
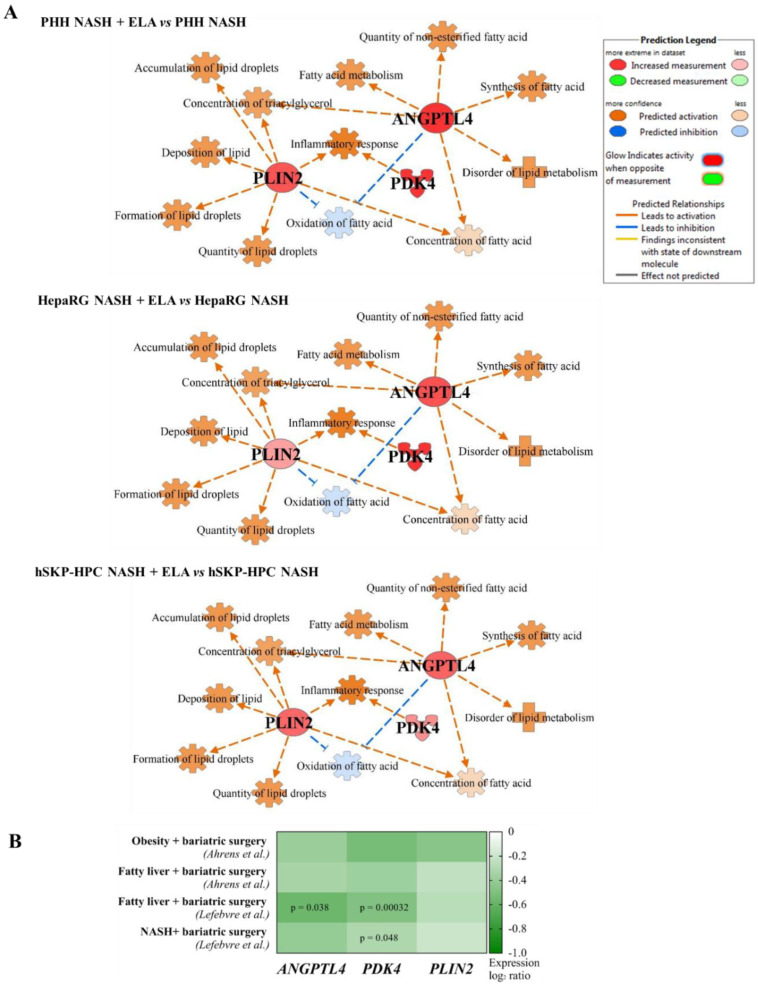
Elafibranor-mediated upregulation of ANGPTL4, PDK4, and PLIN2 is predicted to induce steatosis functions in in vitro NASH models, contrarily to observed downregulation in patients with resolved NASH due to bariatric surgery. (**A**) Regulation of steatosis- and inflammation-related functions by PLIN2, ANGPTL4, and PDK4 in PHH, HepaRG, and hSKP-HPC cultures exposed to ‘NASH’ triggers and elafibranor. (**B**) Differential expression of ANGPTL4, PDK4, and PLIN2 in liver tissue from obesity/NAFLD patients that underwent bariatric surgery.

The robust induction of ANGPTL4, PDK4, and PLIN2 by elafibranor brings up the question of whether or not these lipid metabolism-related genes induce NAFLD-promoting effects. Pathway analysis revealed that these 3 genes have a downstream effect in functions related to hepatic steatosis and inflammation in the three investigated in vitro NASH models (Figure 5A). As such, elafibranor-induced modulation of ANGPTL4, PDK4, and PLIN2 leads to a predicted increase in multiple pro-steatogenic functions, including ‘accumulation and formation of lipid droplets’, ‘fatty acid metabolism’, and ‘concentration of triacylglycerol’. However, the induction of this set of genes is absent in the clinical samples of patients suffering from obesity/fatty liver/NASH that underwent bariatric surgery, which is considered as an intervention that improves NAFLD/NASH (Figure 5B). The discordant effect of elafibranor in in vitro NASH models in comparison with the clinical datasets should be further evaluated as a potential molecular explanation for the failure of elafibranor during the phase 3 ‘RESOLVE-IT’ trial.

## 4. Discussion

NASH is a severe form of NAFLD and is regarded as an important stage during disease progression towards fibrosis, cirrhosis, and cancer. In recent years, several phase 3 NASH clinical trials have failed due to lack of efficacy of the compound under study [20,21]. Therefore, preclinical screening for novel anti-NASH compounds is still highly relevant. However, it is of utmost importance to learn from those failed large clinical studies to improve future pharmacological strategies.

NASH is traditionally studied in animal models. However, due to the complexity of NASH and its human-specificity, NASH-related research has been largely directed towards human-based technologies in recent years [22]. To that extent, we recently developed a methodology that can be employed to study NASH in vitro and screen for potential anti-NASH compounds [6,7]. In the present study, we confirmed the clinical relevance of the thus-developed in vitro disease models by whole genome-based pathway analysis using four publicly available datasets of patients suffering from NASH. Mechanistically, triggering of human hepatic in vitro models resulted in the activation of NASH-specific upstream regulators, including soluble inflammatory mediators and TLRs, as well as transcription factors and kinases known to be activated in the livers of NASH patients, such as NFκB, Jun, and p38 MAPK [23,24]. Furthermore, there were canonical pathways, among which HMGB1- [25] and TREM1-signaling pathways [26] that could be modulated in these in vitro systems, along with inflammation- and cell migration-related processes. Hence, apart for drug screening purposes, these models could also be used for the mechanistic investigation of NASH-promoting processes.

PPAR agonism is considered a promising treatment option for NASH [3]. Elafibranor, a dual modulator of PPAR-α/δ, is one of the developed anti-NASH compounds under evaluation that proved to be effective in a phase 2 study [27]. Nonetheless, an interim analysis of the phase 3 elafibranor trial showed that elafibranor is not more efficacious than placebo [8]. This prompted us to investigate the potential opposite effects to the reduction of NASH characteristics that elafibranor can induce. To this end, we investigated the effects of elafibranor using in vitro NASH models and relied on datasets of patients with resolved NASH upon bariatric surgery as reference. Since no clinical information of elafibranor is available, including a lack of transcriptomic liver data, and since bariatric surgery is the only clinical therapy proven to resolve NASH, among others inducing hepatic metabolic pathways leading to the reduction of liver triglycerides, we believe that these clinical datasets are appropriate to use in our analysis. In a first step, we found that PPARG1A1, PPARA, and SIRT1 are shared elafibranor-induced URs that are predicted as being activated in PHH-, HepaRG-, and hSKP-HPC-derived NASH models. These regulators are, however, known for their NASH-resolving properties, since PPARGC1A1 (i.e., PGC-1α) is a master regulator of mitochondrial biogenesis [28], PPARA expression inversely correlates with histological severity of NASH and recovers upon NASH improvement [29] and deletion of SIRT1, a NAD+-dependent protein deacetylase important for energy homeostasis, results in impaired PPAR-α signaling and decreased fatty acid β-oxidation [30]. In contrast, these three regulators also dictate the increased expression of ANGPTL4, PDK4, and PLIN2, which are known PPAR-α target genes [31]. Although PPAR agonism has been considered as a promising treatment strategy for NASH, these genes are also known for their NASH-promoting effects. Indeed, PLIN2, a lipid droplet protein, promotes obesity and progressive fatty liver disease in mice [32,33], and loss of PLIN2 lessens diet-induced hepatic steatosis, inflammation, and fibrosis [34]. PDK4, which is an inhibitor of the pyruvate dehydrogenase complex [35], is higher expressed on the protein and gene level in NASH liver samples, and PDK4 deficiency in mice leads to reduced steatosis [36]. The effect of ANGPTL4, an exercise-induced hepatokine, is less straightforward. ANGPTL4 is an inhibitor of lipoprotein lipase that stimulates lipolysis but inhibits triglyceride plasma clearance [37]. ANGPTL4 overexpression leads to reduced adiposity at the expense of increased liver steatosis and can therefore be considered as having both positive and negative effects on NASH [38,39]. Notwithstanding, ANGPTL4 has also been earlier proposed as a biomarker for drug-induced steatosis [40].

## 5. Conclusions

By using in vitro NASH models, this study elucidates elafibranor-induced transcriptomic modulations that predict a number of opposing mechanistic effects to those observed in patients with improved NAFLD after bariatric surgery. These ambiguities seem to be related with the modulation of the functions of ANGPTL4, PDK4, and PLIN2 that play prominent roles in lipid metabolism. However, whether these effects truly contributed to the failure of the clinical phase 3 elafibranor trial should be further explored when clinical datasets or samples become available.

## Data Availability

Raw microarray data have been deposited in NCBI’s Gene Expression Omnibus and are accessible through GEO Series accession number GEO: GSE166186. Public available datasets have been obtained as described above under ‘Materials and Methods’.

## Supplementary Materials

The following are available online at https://www.mdpi.com/article/10.3390/cells11050893/s1, Figure S1: Venn Diagram of in vitro NASH disease models, Figure S2: PCA plots of hepatic in vitro models exposed to ‘NASH’ triggers and elafibranor, Figure S3: Modulation inflammatory chemokines in hepatic in vitro models exposed to ‘NASH’ triggers and elafibranor relative to the PHH control samples, Figure S4: Gene expression of TDO2 and ARG2 in human hepatic in vitro models exposed to ‘NASH’ triggers and elafibranor, Table S1: Terms related to Ingenuity Pathway Analysis used in the manuscript.

## References

[1] Cotter T.G., Rinella M. (2020). Nonalcoholic Fatty Liver Disease 2020: The State of the Disease. Gastroenterology.

[2] Younossi Z., Anstee Q.M., Marietti M., Hardy T., Henry L., Eslam M., George J., Bugianesi E. (2018). Global Burden of NAFLD and NASH: Trends, Predictions, Risk Factors and Prevention. Nat. Rev. Gastroenterol. Hepatol..

[3] Boeckmans J., Natale A., Rombaut M., Buyl K., Rogiers V., De Kock J., Vanhaecke T., Rodrigues R.M. (2020). Anti-NASH Drug Development Hitches a Lift on PPAR Agonism. Cells.

[4] Vilar-Gomez E., Martinez-Perez Y., Calzadilla-Bertot L., Torres-Gonzalez A. (2015). Weight Loss via Lifestyle Modification Significantly Reduces Features of Nonalcoholic Steatohepatitis. Gastroenterology.

[5] Klebanoff M.J., Corey K.E., Chhatwal J., Kaplan L.M., Chung R.T., Hur C. (2017). Bariatric Surgery for Nonalcoholic Steatohepatitis: A Clinical and Cost-Effectiveness Analysis. Hepatology.

[6] Boeckmans J., Buyl K., Natale A., Vandenbempt V., Branson S., De Boe V., Rogiers V., De Kock J., Rodrigues R.M., Vanhaecke T. (2019). Elafibranor Restricts Lipogenic and Inflammatory Responses in a Human Skin Stem Cell-Derived Model of NASH. Pharmacol. Res..

[7] Boeckmans J., Natale A., Rombaut M., Buyl K., Cami B., De Boe V., Heymans A., Rogiers V., De Kock J., Vanhaecke T. (2020). Human Hepatic in Vitro Models Reveal Distinct Anti-NASH Potencies of PPAR Agonists. Cell Biol. Toxicol..

[8] (2020). GENFIT Announces Results from Interim Analysis of RESOLVE-IT Phase 3 Trial of Elafibranor in Adults with NASH and Fibrosis.

[9] Harrison S.A., Ratziu V., Bedossa P., Dufour J.-F., Kruger F., Schattenberg J.M., Francque S.M., Arrese M., George J., Bugianesi E. RESOLVE-IT Phase 3 Trial of Elafibranor in NASH: Final Results of the Week 72 Interim Surrogate Efficacy Analysis. Proceedings of AASLD 2020.

[10] Lefere S., Onghena L., Vanlander A., van Nieuwenhove Y., Devisscher L., Geerts A. (2021). Bariatric Surgery and the Liver-Mechanisms, Benefits, and Risks. Obes. Rev..

[11] Chambers A.P., Jessen L., Ryan K.K., Sisley S., Wilsonpérez H.E., Stefater M.A., Gaitonde S.G., Sorrell J.E., Toure M., Berger J. (2011). Weight-Independent Changes in Blood Glucose Homeostasis after Gastric Bypass or Vertical Sleeve Gastrectomy in Rats. Gastroenterology.

[12] Rodrigues R.M., De Kock J., Branson S., Vinken M., Meganathan K., Chaudhari U., Sachinidis A., Govaere O., Roskams T., De Boe V. (2014). Human Skin-Derived Stem Cells as a Novel Cell Source for In Vitro Hepatotoxicity Screening of Pharmaceuticals. Stem Cells Dev..

[13] Rodrigues R.M., Heymans A., De Boe V., Sachinidis A., Chaudhari U., Govaere O., Roskams T., Vanhaecke T., Rogiers V., De Kock J. (2016). Toxicogenomics-Based Prediction of Acetaminophen-Induced Liver Injury Using Human Hepatic Cell Systems. Toxicol. Lett..

[14] Ahrens M., Ammerpohl O., von Schönfels W., Kolarova J., Bens S., Itzel T., Ahrens M., Ammerpohl O., von Scho W., Teufel A. (2013). DNA Methylation Analysis in Nonalcoholic Fatty Liver Disease Suggests Distinct Disease-Specific and Remodeling Signatures after Bariatric Surgery. Cell Metab..

[15] Lake A.D., Novak P., Fisher C.D., Jackson J.P., Hardwick R.N., Billheimer D.D., Klimecki W.T., Cherrington N.J. (2011). Analysis of Global and Absorption, Distribution, Metabolism, and Elimination Gene Expression in the Progressive Stages of Human Nonalcoholic Fatty Liver Disease. Drug Metab. Dispos..

[16] Horvath S., Erhart W., Brosch M., Ammerpohl O., Von Schönfels W., Ahrens M., Heits N., Bell J.T., Tsai P.C., Spector T.D. (2014). Obesity Accelerates Epigenetic Aging of Human Liver. Proc. Natl. Acad. Sci. USA.

[17] Suppli M.P., Rigbolt K.T.G., Veidal S.S., Heebøll S., Eriksen P.L., Demant M., Bagger J.I., Nielsen J.C., Oró D., Thrane S.W. (2019). Hepatic Transcriptome Signatures in Patients with Varying Degrees of Nonalcoholic Fatty Liver Disease Compared with Healthy Normal-Weight Individuals. Am. J. Physiol. Gastrointest. Liver Physiol..

[18] Lefebvre P., Lalloyer F., Baugé E., Pawlak M., Gheeraert C., Dehondt H., Vanhoutte J., Woitrain E., Hennuyer N., Mazuy C. (2017). Interspecies NASH Disease Activity Whole-Genome Profiling Identifies a Fibrogenic Role of PPARα-Regulated Dermatopontin. JCI Insight.

[19] Miura K., Seki E., Ohnishi H., Brenner D.A. (2010). Role of Toll-like Receptors and Their Downstream Molecules in the Development of Nonalcoholic Fatty Liver Disease. Gastroenterol. Res. Pract..

[20] Lambrecht J., van Grunsven L.A., Tacke F. (2020). Current and Emerging Pharmacotherapeutic Interventions for the Treatment of Liver Fibrosis. Expert Opin. Pharmacother..

[21] Neuschwander-Tetri B.A. (2020). Therapeutic Landscape for NAFLD in 2020. Gastroenterol.

[22] Boeckmans J., Natale A., Buyl K., Rogiers V., De Kock J., Vanhaecke T., Robim M. (2018). Human-Based Systems: Mechanistic NASH Modelling Just around the Corner?. Pharmacol. Res..

[23] Frades I., Andreasson E., Mato J.M., Alexandersson E., Matthiesen R., Martínez-Chantar M.L. (2015). Integrative Genomic Signatures Of Hepatocellular Carcinoma Derived from Nonalcoholic Fatty Liver Disease. PLoS ONE.

[24] Schuster S., Cabrera D., Arrese M., Feldstein A.E. (2018). Triggering and Resolution of Inflammation in NASH. Nat. Rev. Gastroenterol. Hepatol..

[25] Gaskell H., Ge X., Nieto N. (2018). High-Mobility Group Box-1 and Liver Disease. Hepatol. Commun..

[26] Rao S., Huang J., Shen Z., Xiang C., Zhang M., Lu X. (2019). Inhibition of TREM-1 Attenuates Inflammation and Lipid Accumulation in Diet-Induced Nonalcoholic Fatty Liver Disease. J. Cell. Biochem..

[27] Ratziu V., Harrison S.A., Francque S., Bedossa P., Lehert P., Serfaty L., Romero-Gomez M., Boursier J., Abdelmalek M., Caldwell S. (2016). Elafibranor, an Agonist of the Peroxisome Proliferator-Activated Receptor-α and -δ, Induces Resolution of Nonalcoholic Steatohepatitis Without Fibrosis Worsening. Gastroenterology.

[28] Scarpulla R.C. (2011). Metabolic Control of Mitochondrial Biogenesis through the PGC-1 Family Regulatory Network. Biochim. Biophys. Acta.

[29] Francque S., Verrijken A., Caron S., Prawitt J., Paumelle R., Derudas B., Lefebvre P., Taskinen M.R., Van Hul W., Mertens I. (2015). PPAR-α Gene Expression Correlates with Severity and Histological Treatment Response in Patients with Non-Alcoholic Steatohepatitis. J. Hepatol..

[30] Purushotham A., Schug T.T., Xu Q., Surapureddi S., Guo X., Li X. (2009). Hepatocyte-Specific Deletion of SIRT1 Alters Fatty Acid Metabolism and Results in Hepatic Steatosis and Inflammation. Cell Metab..

[31] Rakhshandehroo M., Knoch B., Müller M., Kersten S. (2010). Peroxisome Proliferator-Activated Receptor Alpha Target Genes. PPAR Res..

[32] Najt C.P., Lwande J.S., McIntosh A.L., Senthivinayagam S., Gupta S., Kuhn L.A., Atshaves B.P. (2014). Structural and Functional Assessment of Perilipin 2 Lipid Binding Domain(s). Biochemistry.

[33] Orlicky D.J., Libby A.E., Bales E.S., McMahan R.H., Monks J., La Rosa F.G., McManaman J.L. (2019). Perilipin-2 Promotes Obesity and Progressive Fatty Liver Disease in Mice through Mechanistically Distinct Hepatocyte and Extra-Hepatocyte Actions. J. Physiol..

[34] Najt C.P., Senthivinayagam S., Aljazi M.B., Fader K.A., Olenic S.D., Brock J.R.L., Lydic T.A., Jones A.D., Atshaves B.P. (2016). Liver-Specific Loss of Perilipin 2 Alleviates Diet-Induced Hepatic Steatosis, Inflammation, and Fibrosis. Am. J. Physiol.-Gastrointest. Liver Physiol..

[35] Zhang S., Hulver M.W., McMillan R.P., Cline M.A., Gilbert E.R. (2014). The Pivotal Role of Pyruvate Dehydrogenase Kinases in Metabolic Flexibility. Nutr. Metab..

[36] Zhang M., Zhao Y., Li Z., Wang C. (2018). Pyruvate Dehydrogenase Kinase 4 Mediates Lipogenesis and Contributes to the Pathogenesis of Nonalcoholic Steatohepatitis. Biochem. Biophys. Res. Commun..

[37] Takahashi H., Kotani K., Tanaka K., Egucih Y., Anzai K. (2018). Therapeutic Approaches to Nonalcoholic Fatty Liver Disease: Exercise Intervention and Related Mechanisms. Front. Endocrinol..

[38] Mandard S., Zandbergen F., Van Straten E., Wahli W., Kuipers F., Müller M., Kersten S. (2006). The Fasting-Induced Adipose Factor/Angiopoietin-like Protein 4 Is Physically Associated with Lipoproteins and Governs Plasma Lipid Levels and Adiposity. J. Biol. Chem..

[39] Xu A., Lam M.C., Chan K.W., Wang Y., Zhang J., Boo R.L.C., Xu J.Y., Chen B., Chow W.S., Tso A.W.K. (2005). Angiopoietin-like Protein 4 Decreases Blood Glucose and Improves Glucose Tolerance but Induces Hyperlipidemia and Hepatic Steatosis in Mice. Proc. Natl. Acad. Sci. United States Am..

[40] Sahini N., Selvaraj S., Borlak J. (2014). Whole Genome Transcript Profiling of Drug Induced Steatosis in Rats Reveals a Gene Signature Predictive of Outcome. PLoS ONE.

