# Supplementary material for: Transcriptomics Reveals Discordant Lipid Metabolism Effects between In Vitro Models Exposed to Elafibranor and Liver Samples of NAFLD Patients after Bariatric Surgery
Source: Cells. 2022 Mar 4;11(5):893. doi: 10.3390/cells11050893 (PMC8909190; doi:10.3390/cells11050893)
Supplement: Supplementary file 1 [file cells-11-00893-s001.zip › cells-1601778-supplementary.pdf]

Supplementary Materials

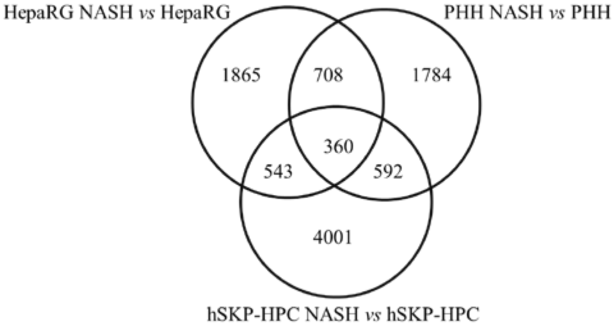

Fig. S1: Venn Diagram of *in vitro* NASH disease models (probesets-based; total number: 54613)

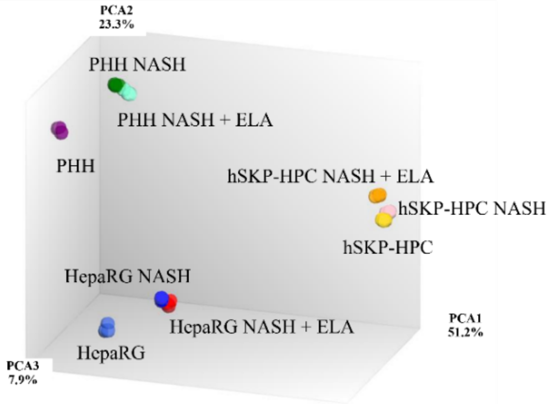

Fig. S2: PCA plots of hepatic *in vitro* models exposed to 'NASH' triggers and elafibranor. [eBayes, $p < 0.05$, fold change < -2 or > +2]

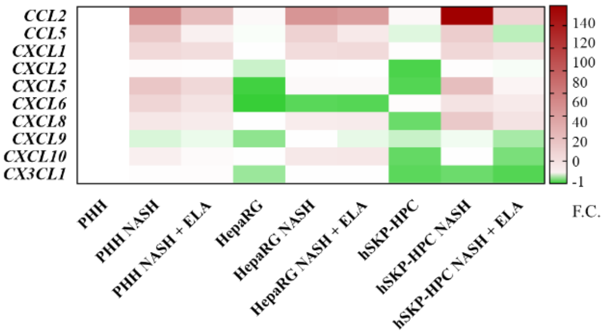

Fig. S3: Modulation inflammatory chemokines in hepatic *in vitro* models exposed to 'NASH' triggers and elafibranor relative to the PHH control samples. (F.C.: fold change)

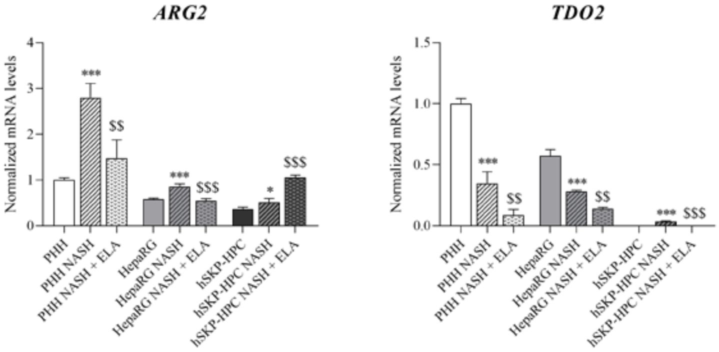

Fig. S4: Gene expression of TDO2 and ARG2 in human hepatic *in vitro* models exposed to 'NASH' triggers and elafibranor. [One-way ANOVA with post hoc Bonferroni correction vs control condition (* and ***; $p \leq 0.05$ and $p \leq 0.001$) and vs 'NASH' condition ($$ and $$$; $p \leq 0.01$ and $p \leq 0.001$)]

Table S1: Terms related to Ingenuity Pathway Analysis used in the manuscript.

Term	Explanation	Source
Diseases & functions	*"Predicts affected biology based on gene expression and predicts directional change on that effect". Diseases & functions can be considered as downstream effects.*	*Joe Salvatore and Dev Mistry, Ingenuity Pathway Analysis (IPA): Maximizing the Biological Interpretation of Gene,*

		Transcript & Protein Expression Data with IPA, 2017.
Overall similarity z-score and associated p-value	*"An average of the four scores computed in Analysis Match: Canonical Pathways (abbreviated in IPA as "CP"), Upstream Regulators (abbreviated "UR" in IPA), Causal Networks (abbreviated "CN" in IPA) and Diseases and Functions (abbreviated "DE" in IPA, also known as Downstream Effects)"*<br>*"Finally, IPA calculates an overall p-value average across the four signatures as follows:*<br>*1. Compute the –log10 of each p-value (if –log10 of p-value is > 50, then set it to 50).*<br>*2. Calculate % vs. the maximum possible p-value (self vs. self).*<br>*3. Report the average of all four percentages."*	*Stuart M. Tugendreich, Understanding biological mechanisms in transcriptomics or proteomics datasets with Ingenuity Pathway Analysis (IPA) and Analysis Match, 2019.*
Upstream regulator	*"The goal of the IPA Upstream Regulator analytic is to identify the cascade of upstream transcriptional regulators that can explain the observed gene expression changes in a user's dataset, which can help illuminate the biological activities occurring in the tissues or cells being studied."*<br>*"The upstream regulator analysis is based on prior knowledge of expected effects between transcriptional regulators and their target genes stored in the Ingenuity® Knowledge Base."*	*Ingenuity Upstream Regulator Analysis in IPA® (www.digitalinsights. qiagen.com)*
Similarity z-score and associated p-value	*= actual z-score divided by the maximum possible z-score*<br>*"To make the results easier to interpret, the z-scores arescaled to a percentage from -100% to +100% by dividing them by the z-score that would result from a perfect match."*<br>*"In addition to the z-score-based metric described above, IPA also independently tests whether the overlap between any two signatures has statistical significance by calculating a p-value. The Fisher's Exact Test is employed."*	*Stuart M. Tugendreich, Understanding biological mechanisms in transcriptomics or proteomics datasets with Ingenuity Pathway Analysis (IPA) and Analysis Match, 2019.*
z-score	*"For each biological function a statistical quantity is computed, called the activation z-score. The activation z-score is used to infer likely activation states of biological functions based on comparison with a model that assigns random regulation directions. Under ideal circumstances (the "un-biased" case described below) the activation z-score can also be used to predict implicated biological functions independently from their associated p-value, based on significant pattern match of up/down regulation."*	*Ingenuity Downstream Effects Analysis in IPA® (www.digitalinsights. qiagen.com)*
